# Changes in Arterial Stiffness in Response to Various Types of Exercise Modalities: A Narrative Review on Physiological and Endothelial Senescence Perspectives

**DOI:** 10.3390/cells11223544

**Published:** 2022-11-09

**Authors:** Sandhya Kresnajati, Yi-Yuan Lin, Toby Mündel, Jeffrey R. Bernard, Hsin-Fu Lin, Yi-Hung Liao

**Affiliations:** 1 Department of Exercise and Health Sciences, National Taipei University of Nursing and Health Sciences, Taipei 11219, Taiwan; 2 School of Sport, Exercise and Nutrition, Massey University, Palmerston North 4474, New Zealand; 3 Department of Kinesiology, California State University-Stanislaus, Turlock, CA 95382, USA; 4 Department of Athletics, National Taiwan University, Taipei 10617, Taiwan; 5 Master Program of Sports Facitility Management and Health Promotion, National Taiwan University, Taipei 10617, Taiwan

**Keywords:** pulse wave velocity (PWV), interval exercise, resistance exercise, aerobic exercise, arterial compliance, insulin sensitivity, endothelial function

## Abstract

Arterial stiffness is a reliable independent predictor of cardiovascular events. Exercise training might enhance arterial compliance through improved metabolic health status. Different modes of exercise may have different effects on arterial stiffness. However, the interactions among different modes of exercise on endothelial senescence, the development of arterial vascular stiffness, and the associated molecular mechanisms are not completely understood. In this narrative review, we evaluate the current evidence focusing on the effects of various exercise modes on arterial stiffness and vascular health, and the known underlying physiological mechanisms are discussed as well. Here, we discuss the most recent evidence of aerobic exercise, high-intensity interval training (HIIT), and resistance exercise (RE) on arterial stiffness and endothelial senescence in physiological and cellular studies. Indeed, aerobic, HIIT, and progression RE-induced arterial compliance may reduce arterial stiffness by effectively promoting nitric oxide (NO) bioavailability and reducing endothelial senescence. However, the transient increase in inflammation and sympathetic activation may contribute to the temporary elevation in arterial stiffness following whole-body high-intensity acute resistance exercise.

## 1. Introduction

Arterial stiffness is a primary factor in cardiovascular disease (CVD), strokes, coronary heart disease, and mortality [1,2]. The development of arterial stiffness is multifaceted as it is a natural physiological response to aging as well as a product of poor lifestyle choices such as lack of physical activity, an unhealthy diet, and smoking [3,4]. As measured by pulse wave velocity (PWV), arterial stiffness is a reliable predictor of cardiovascular morbidity and mortality caused by arterial dysfunction [5]. Central arteries have multiple layers of elastin, whereas peripheral arteries contain more smooth muscle cells. Moreover, arterial architecture and function vary considerably across the arterial tree, and arterial tissue remodeling associated with aging and vascular risk factors also varies across arterial territories. Therefore, to characterize segment-specific PWV is necessary and has been documented in several methodological reviews [6,7,8,9]. Carotid–femoral pulse wave velocity (cfPWV, also known as central PWV) is considered the gold standard measurement of arterial stiffness. The femoral–ankle pulse wave velocity (faPWV) is usually referred to as peripheral PWV. The brachial–ankle pulse wave velocity (baPWV) is a composite of central and peripheral arterial stiffness [10]. Specifically, arterial dysfunction is characterized by the thickening of the arterial wall and a reduction in endothelial and autonomic function [11]. Recently, metabolic disorders/impairments, and the subsequent metabolic syndrome (MetS), have been shown to have a strong association with increased CVD risk in epidemiological studies [12,13,14]. The given evidence reveals strong associations among cardiovascular dysfunction, impairments in metabolic health, and lack of physical activity.

Increased arterial stiffness is an early indicator of the risk of CVD as stiffer vessels predict heart attack and stroke in adults, particularly those with type 2 diabetes mellitus [12,13]. In addition, obese individuals with insulin resistance have a far greater cardiovascular risk profile and stiffer arteries [15]. Impaired glucose regulation, defined by post-challenge hyperglycemia and insulin resistance, plays a significant role in subclinical arterial stiffness in the general population. Furthermore, the triglyceride glucose (TyG) index was found to be significantly associated with arterial stiffness as measured by brachial–ankle pulse wave velocity [16,17]. Hypertension, metabolic syndrome, and aging appear to be tightly associated with increased PWV [18,19,20,21]. However, these above factors lead to negative structural and functional impacts on the vascular system, and the underlying mechanisms for these risk factors vary. Evidence reveals that hypertension causes vascular damage, elastin fragmentation, extracellular matrix changes, and other mechanisms that contribute to hemodynamic overload, which in turn impairs vascular structural remodeling [18]. Hyperinsulinemia and hyperglycemia induce sympathetic activation and vascular inflammatory response due to deterioration of glucose tolerance, leading to the progression of vascular wall hypertrophy and fibrosis. Moreover, accelerated production of glycosylated end-products (AGEs) and cross-linking of collagen and elastin fibers in the arterial wall in the mid- to late-stage reduce vascular compliance [19]. Aging-induced increase in arterial stiffness attributes to collagen deposition, decrease in elastin, and calcification of the vascular wall, resulting in changes in the vascular extracellular matrix, thereby suppressing vascular compliance [20].

It is well established that regular exercise exerts clear protective benefits to enhance overall glycemic control, promote insulin sensitivity, and improve lipid metabolism [22,23]. Thus, indicating that exercise training might enhance arterial compliance through improved metabolic health status. However, there is still little known as to whether the mode of exercise affects arterial stiffness differently on an acute basis. On the other hand, cellular senescence, a process occurring when continuously exposed to cellular stressors, has been considered another primary factor affecting vascular health [24]. Vascular endothelial cell senescence is known to be a major risk factor for CVD [25,26] due to the development of endothelial dysfunction [27]. Moreover, metabolic degenerative factors (e.g., obesity, insulin resistance, and type 2 diabetes) can cause endothelial morphological changes and endothelial dysfunction, leading to arterial stiffness, atherosclerosis, hypertension, stroke, and coronary artery disease [28,29,30,31]. However, there are still rare investigations focusing on the impacts of lifestyle factors (e.g., habitual physical exercise) on endothelial senescence and arterial stiffness.

In healthy and diabetic populations, acute aerobic exercise effectively reduces central arterial stiffness, wave reflections, and hemodynamics [32,33,34,35]. While acute bouts of resistance exercise may cause transient increases in central arterial stiffness [36], this is not a universal finding. Because previous studies have not been consistent, more research is needed to assess the effects of various exercise modes on arterial stiffness. The overarching goals for this narrative review were to determine the: (1) potential factors (i.e., metabolic disorder, obesity, endothelial cell senescence, physical exercise) influencing arterial stiffness and the risks for future cardiovascular events; (2) effect of aerobic exercise on arterial stiffness; (3) effect of resistance exercise on arterial stiffness; and (4) the effect of interval exercise, particularly high-intensity interval training (HIIT), on arterial stiffness. Thus, the current literature on the effects of aerobic exercise, resistance exercise, and interval exercise on changes in arterial stiffness and the potential underlying physiological/molecular mechanisms have been compiled in this narrative review.

## 2. Data Sources and Search Strategies

An extensive search of PubMed/MEDLINE, Web of Science (WOS), and Google Scholar for articles published in English databases from the database’s inception in 2000 through December 2021 were conducted. Literature searches were restricted to studies that involved exercise and the materials were written in English. For simplicity, we used standardized terms rather than phrases. Keywords used in the searches were: HIIT, high-intensity interval training, HIIE, high-intensity interval exercise, interval exercise, resistance exercise, resistance training, aerobic exercise, aerobic training, arterial stiffness, insulin resistance, HOMA-IR, endothelial function, and endothelial senescence. Search strategies were customized for each database. We used the Boolean operators “OR” and “AND” to search all descriptors to ensure the most comprehensive search possible. After we used feature sort from the databases and reviewed each article abstract, the randomized control or clinical research types were first considered and included in the summary tables in this narrative review. Furthermore, eligible studies focusing on the physiological and molecular mechanisms that mediated the benefits of exercise were found in the reference lists of relevant articles and reviews found during the searches. Database alerts for recently published studies were also constantly checked for new and potentially eligible studies.

## 3. Potential Factors Influencing Arterial Stiffness and Cardiovascular Health

Arterial stiffness is often quantitated by the velocity an arterial waveform travels between different locations in the arterial territory. Despite the methods to quantitate arterial stiffness being beyond the scope of the current review, pulse wave velocity between cfPWV and baPWV are widely used surrogates of central and systemic arterial stiffness in clinical settings; faPWV or heart-to-radial (hrPWV) pulse wave velocity is often used to access peripheral artery stiffness in the literature. Other measures of arterial structure remodeling such as carotid arterial compliance and carotid intima–media thickness (cIMT) assessed by the use of ultrasonography are available to characterize central arterial stiffness. Arterial waveform decomposition analysis also provides significant clinical information on systemic arterial stiffening. To gain more insights, there are some valuable methodological reviews [6,7,8,9] for reference in the literature.

Epidemiological evidence revealed that the prevalence of cardiovascular risk factors and metabolic disorders increased dramatically with age. Furthermore, this association of metabolic impairments with future cardiovascular events was most evident in populations younger than 60 years of age [14]. Similar associations have been reported in epidemiological studies in both developed [13] and developing countries [4]. Taken together these findings suggest that metabolic impairments can have a negative impact on public health, particularly cardiovascular health. Previous studies have shown that the development of metabolic disorders, and subsequently MetS, were associated with increased cardiovascular system damage and accelerated age-related arterial changes [3], such as arterial stiffness, as assessed mainly by aortic PWV [9,37], cardio–ankle vascular index (CAVI) [38], etc. Moreover, cumulative studies have reported aortic PWV as an independent predictor of total mortality and future cardiovascular events [39,40,41]. Therefore, in this narrative review, we also focused on studies assessing arterial functional measurements using these gold standards to reflect cardiovascular health status, and the reports focusing on the association between arterial stiffness and metabolic impairments/disorders are deeply discussed as well. Summary of the associations between metabolic impairments and arterial stiffness are shown in Table 1.

The conceptualization of metabolic disorder focused on a central role of insulin resistance development and this is associated with CVD and defined as the circumstances of obesity that are mutually associated with hypertension, hypertriglyceridemia, impaired glucose tolerance, decrease HDL cholesterol, and abdominal obesity [42]. Using International Society of Vascular Health and Aging patients enrolled in 32 centers from 18 European countries it was found that MetS and age have different effects on the CAVI, another systemic stiffness index, and cfPWV, with age having a more pronounced impact on CAVI and MetS increasing cfPWV but not CAVI [38]. Scuteri et al. studied 20,570 subjects from nine MARE Consortium cohorts representing eight different European countries and the United States and showed that MetS clusters are consistently associated with significantly stiffer arteries to the same or greater extent as subjects with changes in age, gender, smoking, cholesterol levels, and diabetes mellitus [43]. In addition to the above studies, several clinical studies have evaluated the effects of decreased metabolic fitness and changes in hemodynamic parameters on arterial health. Thus, the relationships between decreased metabolic health (e.g., glucose intolerance, dyslipidemia, central obesity, etc.) and the development of arterial stiffness in the aorta and other large arteries have been well documented [37,44].

One of the mechanisms linking insulin resistance and CVD may be increased arterial stiffness [3,45]. PWV is an index of arterial stiffness and insulin resistance represented by the homeostasis model assessment of insulin resistance (HOMA-IR) with varying fasting glucose levels [2]. Ryder et al. investigated the association of insulin resistance and obesity with flow-mediated dilation (FMD), cIMT, and arterial stiffness in children [46]. The findings revealed that obesity, visceral adipose tissue (VAT), and IR were all significantly associated with cIMT (*p* < 0.05). Another previous study found that insulin resistance index, measured by HOMA-IR, exhibits a good correlation (range: r = 0.41–0.55 in both genders) with several lipid metabolic biomarkers (i.e., triglyceride glucose index, triglyceride to high-density lipoprotein cholesterol ratio, visceral adiposity index, and lipid accumulation product), and all above variables were positively correlated with increased baPWV in both sexes (*p* < 0.01) [47]. All surrogate insulin markers demonstrated an excellent ability to predict high baPWV concerning HOMA-IR, suggesting that insulin resistance plays a critical role in the development of arterial stiffness. Of note, hypertension is believed to be a strong regulator of arterial stiffness [21]. However, some research evidence has demonstrated that arterial stiffness increased independent of blood pressure in MetS patients [8,48,49]. Although the studies using correlation analyses might not directly explain the causal effect and possible consequence, these findings certainly reveal the potentially deleterious impacts of metabolic impairments on the development of arterial stiffness and future cardiovascular events.

On the other hand, Hughes et al. discovered that insulin reduction and weight loss were associated with decreases in baPWV (1207.6 ± 132.3 cm/s) (*p* < 0.001) but not faPWV (945.9 ± 102.8 cm/s) (*p* = 0.385) or cfPWV (880.0 ± 257.4 cm/s) (*p* = 0.046). In addition, this study found that both weight loss and insulin reductions had a direct and positive effect on arterial stiffness [48]. Fantin et al. discovered that cfPWV was related to glycemia, triglycerides, and the HOMA index. In this study, researchers found a link between neck circumference, insulin resistance, and arterial stiffness in a group of overweight and obese people [49]. Sengstock et al. showed that insulin sensitivity is inversely related to arterial stiffness in hypertensive older adults without diabetes [50]. The above studies further suggest that applying appropriate approaches to ameliorate metabolic health status (e.g., reducing body weight, lowering central obesity, and enhancing insulin sensitivity and glycemic control) could help to attenuate the development of arterial stiffness in varied populations.

Cellular senescence is a process that occurs naturally in cells as we age due to continuous exposure to cellular stressors [24], which has been considered another primary factor affecting vascular health. Vascular EC senescence is known to be a major risk factor for CVD [25,26]; moreover, the increased risk of CVD with age is mainly a consequence of the development of endothelial dysfunction [27]. The vascular endothelium, a single layer of cells adjacent to the lumen, plays an important physiological role in vascular homeostasis, including maintaining blood flow, regulating vascular tone, regulating the production of pro-inflammatory molecules, and promoting vascular neovascularization [28]. In addition to aging factors, metabolic degenerative factors such as obesity, insulin resistance, and type 2 diabetes can cause endothelial morphological changes and endothelial dysfunction, leading to arterial stiffness, atherosclerosis, hypertension, stroke, and coronary artery disease [28,29,30,31]. To date, many lines of evidence suggest that aging may have deleterious effects on vascular EC function and that vascular EC senescence plays a key role in the development, progression, and progression of vascular aging leading to CVD [51,52,53,54,55,56,57,58]; for example, aging vascular ECs exhibit reduced production of the key vasodilatory molecule nitric oxide (NO) [51,52,53], increased production of reactive oxygen species [54,55], increased release of endothelin-1 (ET-1) [56], and stress-induced apoptosis [58], all of which lead to vasodilatory dysfunction and dysregulation of arterial compliance. Moreover, some of the cellular biomarkers associated with cellular senescence have also been identified to be associated with decreased vascular EC function. Increased expression of p21 and p16 in the arterial tissue of aged mice has been reported to be associated with oxidative stress-mediated inhibition of NO-dependent vascular endothelial function [57]. Thus, the aforementioned cellular senescence factors related to aging or non-aging directly (e.g., obesity, metabolic impairment, systemic inflammation, etc.) cause a decrease in normal vascular EC function, which further leads to impaired vasodilatation and reduced blood pressure regulation, and subsequently to the development of arterial stiffness. However, the interaction between lifestyle factors (e.g., habitual exercise) on endothelial senescence and the development of arterial vascular stiffness and the associated molecular mechanisms is not yet understood.

Regular exercise improves insulin sensitivity in healthy people and people with lifestyle diseases (such as type 2 diabetes, hypertension, hyperlipidemia, and ischemic coronary artery disease) [59]. Regular physical activity and exercise, particularly endurance exercise, have been proven to enhance cardiovascular functions, thereby decreasing the prevalence of CVD and the mortality of related complications during advancing aging [60,61,62]. Higher physical conditioning status is associated with lower arterial stiffness in a healthy sedentary population and endurance-trained older men compared to their less active peers [63]. Moreover, regular exercise training has clear protective benefits in enhancing overall glycemic control capacity, promoting insulin sensitivity, and improving lipid metabolism [22,23]. Endurance training has also been reported to attenuate the deleterious changes in blood pressure and vascular functions, including the increase in arterial compliance and the decrease in arterial stiffness [64,65]. Several previous studies on the effect of regular exercise on the degree of arterial stiffness suggested that to obtain the benefits of regular exercise on the reduction in arterial stiffness, they should participate in at least two exercise sessions per week [66]. Additionally, it has been suggested that more time spent in physical activity may be helpful in the prevention of arterial stiffness [67], especially since the amount of high-intensity activity involved may also reduce arterial stiffness [68].

However, in healthy people, acute aerobic exercise effectively reduces central arterial stiffness, wave reflections, and hemodynamics [32]. Acute bouts of resistance exercise (three sets of ten repetitions at 75% 1-RM free-weight exercise) may cause a transient increase in central arterial stiffness [36]. Hasegawa et al. (2018) used human and animal models to investigate the effects of different exercise training modalities on vascular health function. In animal studies, HIIT (14 reps of a 20 s swim session with a 10 s interval between sessions, 4 days/week for 6 weeks) and aerobic training (AT; treadmill running, 60 min, 30/min, 5 days/week for 8 weeks) exerted significant benefits on the decrease in aortic pulse wave velocity (PWV) and the exercise-induced decreases in aortic PWV and increase in arterial endothelial nitric oxide synthase/protein kinase B (eNOS/PKB); however, resistance training did not significantly alter these parameters [69]. On the other hand, in the human model, HIIT and AT promote a significant decrease in cfPWV and an increase in plasma nitrite/nitrate levels compared to the sedentary control; moreover, the study further demonstrated that HIIT can reduce central arterial stiffness by increasing NO bioavailability in the aorta and that HIIT can achieve comparable effects to AT despite spending relatively short times exercising [69]. To our knowledge, however, there is still a lack of integrative information focusing on how exercise affects arterial stiffness and whether different exercise type contributes variably to the impacts on arterial compliance. Therefore, in this narrative review, we further evaluate the current evidence focusing on the effects of varied exercise modes on arterial stiffness and vascular health, and the known underlying physiological mechanisms are discussed as well.

**Table 1:** Summary of the associations between metabolic impairments and arterial stiffness.

Authors	Subject	Research Design	Sample Size (*n*)	Assessment Variable	Result/Outcomes
Ho et al., 2010 [45]	Healthy older adults Age: 40 years and above	A population-based prospective cohort study with a stratified, two-stage random sampling approach was used	2188 subjects (Male: 1063 and Female: 1125)	- baPWV - HOMA-IR	**↑ HOMA → ↑ BaPWV (Male and Female)** - HOMA-IR I (1.00 (reference)) [n.s.] - HOMA-IR II (1.15 (0.77–1.71)) [n.s.] - HOMA-IR III (1.60 (1.05–2.46)) [*p* < 0.05]
Webb et al., 2010 [16]	Healthy older adults (risk of diabetes mellitus) Age: ±59 years	A population-based prospective cohort study-screen-detected type 2 diabetes mellitus.	570 subjects (Male: 319 and Female: 251)	- cfPWV - HOMA-IR	**cfPWV mean ± SE** - NGM vs. IGR (9.15 ± 0.12 vs. 9.76 ± 0.11 m/s) [p ≤ 0.001] - NGM vs. DM (9.15 ± 0.12 vs. 9.89 ± 0.22 m/s) [*p* < 0.001] **cfPWV: IGR vs. DM. [n.s.]** **cfPWV → isolated FPG** - FPG vs. NGM (9.77 ± 0.12 vs. 9.15 ± 0.12 m/s) [*p* < 0.001] - 2-HPG vs. NGM (9.95 ± 0.22 vs. 9.15 ± 0.12 m/s) [*p* < 0.001] **HOMA-IR → cfPWV [*p* < 0.01]**
Urbina et al., 2011 [15]	Healthy young adults Age: 15–28 years	A large longitudinal school-based study of the effect of obesity on the development of diabetes	343 subjects (Male: 161 and Female: 182)	- BrachD - PWV - HOMA index	**HOMA index [*p* ≤ 0.0001] (higher = stronger)** - Lean (2.53 ± 0.89) - Obese (2.84 ± 0.73) - Obese IR (7.83 ± 4.02) **Arterial stiffness by obesity and IR.** **AIx (%) [*p* ≤ 0.05] (higher = stiffer)** - Lean (−0.48 ± 11.31) - Obese (0.48 ± 9.04) - Obese IR (3.45 ± 11.73) **BrachD (% change/mmHg) [*p* ≤ 0.0001] (lower = stiffer)** - Lean (6.53 ± 1.21) - Obese (5.71 ± 1.10) - Obese IR (5.47 ± 1.02) **PWV (m/s) [*p* ≤ 0.0001] (higher = stiffer)** - Lean (5.85 ± 0.85) - Obese (6.61 ± 0.99) - Obese IR (6.51 ± 1.21)
Won et al., 2018 [17]	Healthy older adults Age: ±60 years	This is a cross-sectional investigation analyzing baseline data collected for a prospective cohort study	2560 subjects Male: 842 and Female: 1718	- baPWV - TyG index	**↑ baPWV → ↑ TyG index [*p* < 0.001]** - **Group I (lowest)** TyG index (8.7 ± 0.2) baPWV (1421 ± 242 cm/s) - **Group II** TyG index (9.2 ± 0.1) baPWV (1480 ± 244 cm/s) - **Group III** TyG index (9.5 ± 0.1) baPWV (1534 ± 260 cm/s) - **Group IV (highest)** TyG index (10.0 ± 0.3) baPWV (1575 ± 279 cm/s)
Nakagomi et al., 2019 [47]	Healthy middle-aged adults Age: 38.75 ± 9.75 years	This was a cross-sectional study that enrolled non-industrial workers in Japan	2818 subjects (Male: 1720 and Female: 1098)	- TG/HCL-C - VAI, LAP - TyG index - HOMA-IR - baPWV	- **baPWV (cm/s) → insulin resistance markers [*p* < 0.01]** - HOMA-IR (men r = 0.11, women r = 0.14) - TyG index (men r = 0.23, women r = 0.35) - TG/HCL-C (men r = 0.14, women r = 0.29) - VAI (men r = 0.15, women r = 0.30) - LAP (men r = 0.22, women r = 0.34)
Ryder et al., 2016 [46]	Healthy young children Age: 15.1 ± 2.4	This is a cross-sectional study with 2 longitudinal studies conducted at the University of Minnesota	252 subjects (Male: 121 and Female: 131)	- hyperinsulinemic-euglycemic clamp - baFMD - cIMT - PWV	- FMD was positively associated with high adiposity (body mass index, body fat percentage, and VAT) [*p* < 0.01] - Insulin resistance was not associated with FMD. cIMT was significantly, and positively related to obesity, VAT, and insulin resistance [*p* < 0.05] - No differences in carotid incremental elastic modulus and pulse wave velocity [n.s.]
Hughes et al., 2012 [48]	Healthy middle-aged adults Age: 20–45 years	A randomized controlled trial examining the effects of physical activity and weight reduction on improving vascular health	339 subjects (Male: 78 and Female: 261)	- cfPWV - faPWV - baPWV - HOMA-IR	The measures of baseline arterial stiffness were significantly correlated with one another. - HOMA-IR (3.6 ± 2.1) [*p* = 0.045] - baPWV (1207.6 ± 132.3 cm/s) [*p* < 0.001] - faPWV (945.9 ± 102.8 cm/s) [*p* = 0.385] - cfPWV (880.0 ± 257.4 cm/s) [*p* = 0.046]
Fantin et al., 2017 [49]	Overweight/Obese middle-aged adults Age: 20–77 years	A randomized control trial-subject randomly selected by outpatients in the nutritional service of Verona hospital	95 subjects (Male: 42 and Female: 53)	- cfPWV - crPWV - HOMA index - waist, hip, and neck circumference	Subjects with high values of neck circumference had higher insulin resistance. - HOMA (5.09 ± 3.35 vs. 3.66 ± 3.29) [*p* < 0.05] - cfPWV (11.22 ± 2.48 vs. 10.22 ± 1.88 m/s) [*p* < 0.03] - crPWV(10.06 ± 1.57 vs. 9.18 ± 1.43 m/s) [*p* < 0.01]

Abbreviations: 2-HPG, plasma glucose concentration 2 h after a 75 g OGTT; AIx, augmentation index; baPWV, brachial–ankle pulse wave velocity; baFMD, brachial artery flow-mediated dilation; BMI, body mass index; BrachD, brachial artery distensibility; cfPWV, carotid–femoral pulse wave velocity; cIMT, carotid intima–media thickness; crPWV, carotid–radial pulse wave velocity; DM, diabetes mellitus; faPWV, femoral–ankle pulse wave velocity; FMD, flow-mediated dilation; FPG, fasting plasma glucose; HOMA, homeostasis model assessment; HOMA-IR, homeostasis model assessment—insulin resistance; IFG, impaired fasting glucose; IGR, impaired glucose regulation; IR, insulin resistance; LAP, lipid accumulation product; NGM, normal glucose metabolism; PWV, pulse wave velocity; TG/HCL-C, triglyceride to high-density lipoprotein cholesterol ratio; TyG, triglyceride glucose; VAI, visceral adiposity index; VAT, visceral adipose tissue; **→**, correlation/association; **↑**, increase; n.s.; non-significant.

## 4. Effect of Aerobic Exercise on Arterial Stiffness

Aerobic exercise does not require specific equipment and space requirements, and is a simple and convenient exercise modality; in addition, aerobic exercise has considerable benefits to the cardiopulmonary and circulatory systems, and can also produce significant cardiovascular health benefits. Moreover, epidemiological evidence reveals that the increases in aerobic capacity and physical training volume are connected to a lower risk of CVD and mortality [70,71]. However, the prescription patterns for aerobic exercise are more diverse, and less attention has been paid to the acute cardiovascular effects; furthermore, conflicting results have been reported regarding the acute cardiovascular response to aerobic exercise. The effects of aerobic exercise training on arterial stiffness were summaried in Table 2. Pedralli et al. (2020), using FMD to assess endothelial function, reported that 8 weeks of aerobic training (40 min twice a week at 50–75% heart rate reserve) improved endothelial function by 3.2% above baseline in individuals with pre-hypertension or hypertension [72]. A previous study sought to demonstrate that both continuous exercise (6.5 ± 0.1 vs. 5.5 ± 0.2 at baseline and 0 min after exercise) and interval intensity exercise (6.7 ± 0.1 vs. 5.6 ± 0.2 at baseline and 0 min respectively) could reduce systemic arterial stiffness from baseline and post-measurement in healthy young men [33]. Siasos et al. showed that continuous moderate-intensity aerobic exercise (CAE) and high-intensity interval aerobic exercise (hIAE) could both improve endothelial function, implying that acute exercise had an additional cardioprotective effect. Nevertheless, the effect aerobic exercise had on central and peripheral arterial stiffness differs. After exercise, the FMD was reduced for CAE (6.37 ± 1.48 vs. 8.57 ± 2.55%, *p* < 0.001) and also hIAE (5.95 ± 1.78 vs. 8.48 ± 2.60%, *p* < 0.001). In contrast, the study showed no significant difference for cfPWV among both exercises, but only hIAE but not CAE decreased significantly femoral dorsalis pedis pulse wave velocity (fdPWV) after exercise (*p* < 0.001) [34]. An earlier study by Guimarães et al. (2010) compared the effect of continuous vs. interval intensity exercise on arterial stiffness and blood pressure in treated hypertensive patients [73]. Although it was reported that both continuous and interval intensity exercise training were beneficial for controlling blood pressure, only interval intensity training (9.44 ± 0.91 to 8.90 ± 0.96 m/s, *p* = 0.009) reduced arterial stiffness in treated hypertensive subjects [73]. The above findings indicate that aerobic-based interval exercise should have distinct benefits from continuous aerobic exercise mode on improving peripheral/central arterial compliance after exercise. Therefore, we will further focus on the effects of interval or intermittent type exercise on cardiovascular responses in the other section of this review.

Metabolic impairments in poor glycemic control and dyslipidemia have been demonstrated to be significantly associated with arterial stiffness [16,17], and more recent evidence shows that metabolic disorders might blunt the benefits of acute aerobic exercise on reduced arterial stiffness [32]. A study by Way and colleagues (2021) reported that, in diabetic participants, an acute bout of moderate-intensity continuous exercise (MICE; 33 min of cycling at 60–70%HRpeak) decreases the augmentation index at 75 bpm (AI_X_ @75), which reflects the degrees of arterial stiffness by measuring blood pulse-wave reflection, whereas MICE failed to markedly decrease cfPWV after exercise compared to baseline value [32]. Such findings also reveal that moderate intensity of continuous aerobic exercise could only improve arterial compliance to a certain degree (e.g., only exhibited the changes in AIx but not cfPWV) in participants with metabolic disorders.

On the other hand, although the beneficial effects of aerobic exercise on arterial stiffness had been well documented, whether there are gender differences in aerobic exercise-reduced arterial stiffness remained debatable. With this in mind, Doonan et al. evaluated sex differences in arterial stiffness at rest and in response to acute physical stress [35]. They found that carotid–femoral pulse wave velocity (cf-PWV) was significantly higher in men (6.0 ± 0.7 m/s vs. 5.6 ± 0.6 m/s, *p* = 0.03) at rest and at all post-exercise time points compared to women; moreover, the heart rate-adjusted augmentation index was also significantly lower (−10.7 ± 10.2 vs. −4.0 ± 10.9, *p* < 0.0001) in the men [35]. These findings revealed that young men and women may have different arterial compliance characteristics at rest and after acute exercise challenge stress.

From physiological and cellular perspectives, the existing evidence suggests that acute improvements in endothelial function influence reductions in arterial stiffness in humans, which may be mediated primarily by increases in endothelial shear stress experienced during exercise [74]. Regular aerobic exercise may preserve endothelial function with advancing age [75] and reduce the risk of future CVD [70,71], and the specific cellular molecular mechanisms for these chronic benefits may be achieved by slowing the development of cellular senescence. Rossman et al. (2017) examined whether endothelial senescence increases with chronic sedentary behavior and is associated with endothelial dysfunction in a cross-sectional study. The authors found that the expression of p53 (a cellular senescence transcription factor) and the cell cycle protein-dependent kinase inhibitors p21 and p16 in vascular ECs were negatively correlated with endothelial function (brachial artery flow-mediated dilatation), suggesting that endothelial senescence is associated with endothelial dysfunction development [76]. Moreover, the study also showed marked increases in p53, p21, and p16 expression in the endothelium of sedentary older adults (mean 60 years) and younger sedentary individuals (mean 22 years), but similar senescence-related changes in p53 and p21 protein expression in vascular ECs were not observed in older adults (mean 59 years) with habitual exercise; these data suggest that aerobic exercise might suppress endothelial senescence and be considered as a potential intervention to prevent endothelial dysfunction during aging [76].

Similar anti-endothelial senescence effects of exercise were also reported in several animal studies. Werner and colleagues demonstrated that three-week voluntary running-wheel exercise increased telomerase activity in the thoracic aorta tissue of C57/Bl6 mice and decreased the expression of apoptosis regulator proteins (e.g., cell cycle checkpoint kinase 2, p16, and p53) compared to sedentary controls; moreover, voluntary running-trained mice exhibited significantly reduced lipopolysaccharide-induced apoptosis in aortic ECs [58]. The results suggest that regular exercise training modulates vascular tissue telomere stabilizing proteins and reduces cellular senescence biomarkers in mice, thereby preventing stress-induced apoptosis and maintaining endothelial function [58]. Furthermore, mice consuming high-fat fast food diet (FFD; 40% fat, for 16 weeks) significantly increased the expression of p16 and other senescence markers (e.g., p53 and p21 and SA-β-gal activity) in adipocytes, yet exercise training (wireless running wheels) reduced the expression of these cellular senescence markers in visceral adipose tissue [77], indicating that exercise may provide restorative benefits by reducing the accumulated cellular senescence burden. Based on the above evidence on endothelial senescence, aging, prolonged sedentary lifestyle, and unhealthy dietary patterns may upregulate p53, p21, and p16 ^Ink4a^ expression and cellular senescence responses in vascular ECs or other metabolic-related tissues [60,78,79], but these negative changes appear to be mitigated or prevented through exercise training. However, it is still not clear how aerobic exercise modulates the aging of vascular EC with advancing age. Although several investigations have recently made progress in identifying the mechanisms underlying endothelial senescence and the potential benefits of exercise training, the potential benefits and molecular mechanisms involving diverse types of exercise modalities induced are still unclear due to the complexity of exercise prescription parameters. Taken together, aerobic exercise causes a transient improvement in arterial stiffness by improving endothelial function and suppressing endothelial senescence, implying another cardiovascular protective effect.

**Table 2:** Effect of aerobic exercise training on arterial stiffness.

Authors	Subject	Research Design	Sample Size	Intervention	Intensity	Assessment Variable	Result/Outcomes
Wang et al., 2014 [33]	Healthy young male students Age: 21.2 ± 0.4 years	A randomized balanced self-control crossover design was used in this study	15 subjects	CE (Continuous Exercise) IE (Interval Exercise) Cycling Ergometer	30 min at 35% HRR and 15-min separated by a 20-min rest	CAVI Measured at: - Baseline (BL) - 0 min postEx - 40 min postEx	The time-dependent changes in CAVI were significantly different between the control and intervention groups. **CON trial** - BL (6.7 ± 0.1) - 0 min (6.7 ± 0.1) ⟷ - 40 min (6.6 ± 0.1) ⟷ **CE trial** - BL (6.5 ± 0.1) - 0 min (5.5 ± 0.2) ↓ - 40 min (6.4 ± 0.1) ⟷ **IE trial** - BL (6.7 ± 0.1) - 0 min (5.6 ± 0.2) ↓ - 40 min (6.0 ± 0.1) ↓
Siasos et al., 2016 [34]	Healthy young men Age: 22.6 ± 3.3 years	This study used a cross-over study design	20 subjects	CAE (Intensity Aerobic Exercise) hIAE (High-Intensity Interval Aerobic Exercise) Cycling Ergometer	30 min at 50% of maximum aerobic work	- FMD - cfPWV - fdPWV Measured at: - 10 min preEx - 10 min postEx	**FMD** - CAE (6.37 ± 1.48 vs. 8.57 ± 2.55%) [*p* < 0.001] - hIAE (5.95 ± 1.78 vs. 8.48 ± 2.60%) [*p* < 0.001] **cfPWV** - CAE (5.87 ± 0.82 vs. 5.76 ± 0.63 m/s−1) [*p* = 0.27] - hIAE (5.87 ± 0.67 vs. 5.80 ± 0.57 m/s−1) [*p* = 0.40] **fdPWV** - CAE (9.27 ± 1.11 vs. 8.17 ± 1.48 m/s−1) [*p* < 0.003] - hIAE (9.14 ± 1.07 vs. 8.26 ± 0.8 m/s−1) [*p* < 0.001]
Doonan et al., 2013 [35]	Healthy young adults Age: 24.05 ± 5.5 years	This study used a cross-sectional study design	122 subjects	Aerobic Exercise Treadmill Running	Exercise protocol to volitional exhaustion (sprint)	- AIx75 - SEVR - cfPWV Measured at: - 10 min preEx - 2 min postEx - 5 min postEx - 10 min postEx - 15 min postEx	- **cfPWV (m/s^−1^)** (6.0 ± 0.7 vs. 5.6 ± 0.6) - **AIx75 (%)** (10.7 ± 10.2 vs. 4.0 ± 10.9) - **SEVR (%)** (176.2 ± 43.8 vs. 163.4 ± 40.9)
Way et al., 2021 [32]	Diabetes adult patients VO_2peak_: 25.2 ± 1.1 mL/min/kg Age: 29–59 years	This study used a randomized cross-over design	24 subjects	HIIE (High-Intensity Interval Training) MICE (Moderate-Intensity Continuous Exercise) Cycling Ergometer	- HIIE: cycling for 4 × 4 min at 85–95% of HRpeak. - MICE: 33 min of continuous cycling at 60–70% HRpeak. - CON: lying quietly in a supine position for 30 min	- cfPWV - Aix - AIx75 Measured at: - 30 min preEx - 0 min postEx - 30 min postEx - 60 min postEx	**cfPWV (m/s) [n.s]** - HIIE Group (8.1 ± 0.2, 8.1 ± 0.2, 7.9 ± 0.2, 8.0 ± 0.2) - MICE Group (8.2 ± 0.3, 8.3 ± 0.4, 8.1 ± 0.2, 8.3 ± 0.3) - CON Group (10.2 ± 2.2, 10.1 ± 2.0, 10.3 ± 2.2, 10.5 ± 2.1) **AIx (%) [n.s]** - HIIE Group (24.5 ± 1.7, 19.9 ± 2.0, 21.1 ± 2.0, 19.0 ± 2.1) - MICE Group (24.5 ± 2.1, 25.0 ± 2.4, 26.9 ± 1.8, 24.8 ± 1.9) - CON Group (26.0 ± 2.3, 22.4 ± 2.4, 24.2 ± 1.7, 24.3 ± 1.9) **AIx75 (%) [*p* = 0.04]** - HIIE Group (24.5 ± 1.7, 26.5 ± 2.0, 24.0 ± 1.8, 18.3 ± 2.2) - MICE Group (27.2 ± 1.8, 25.3 ± 2.2, 25.3 ± 1.8, 21.8 ± 2.1) - CON Group (24.4 ± 2.3, 20.0 ± 2.0, 20.7 ± 1.7, 20.3 ± 1.8) [all above showed the values at 30 min preEx, 0 min postEx, 30 min postEx, and 60 min postEx, respectively]

Abbreviations: AIx, Augmentation index; AIx75, Augmented index in 75 percent; baPWV, brachial–ankle pulse wave velocity; BL, baseline; BP, blood pressure; CAVI, cardio–ankle vascular index; CAE, continuous moderate-intensity aerobic exercise; cfPWV, carotid–femoral pulse wave velocity; CON, control; cSBP, central systolic blood pressure; faPWV, femoral–ankle pulse wave velocity; fdPWV, femoral dorsalis pedis pulse wave velocity; FMD, flow-mediated dilation; HIIT, high-intensity interval training; hIAE, high-intensity interval aerobic exercise; HIIE, high-intensity interval exercise; HR, heart rate; HRmax, heart rate maximum; HRR, Heart rate reserve; IMT, intima–media thickness; LRE, low-intensity resistance exercise; MCT, moderate continuous training; MICE, moderate-intensity continuous exercise; PWV, pulse wave velocity; SEVR, subendocardial viability ratio; T2D, type 2 diabetes; WBV, whole-body vibration; ⟷, no change; ↓, decrease.

## 5. Effect of Resistance Exercise on Arterial Stiffness

From the perspective of CVD and premature death, the existing evidence suggests that physically active individuals present better vascular health, and the benefits of aerobic exercise on cardiovascular health have been well documented in the literature [78,79,80,81,82]. Although, several previous studies have shown that high-volume, low-intensity resistance training is also successful in reducing vascular stiffness [83,84]. However, most studies on resistance exercise focus more on skeletal health, muscle strength, or metabolic health outcomes; investigations directly assessing the impacts of resistance exercise on cardiovascular health remain limited and inconsistent [85,86,87]. Also, acute resistance exercise may cause a transient increase in central arterial stiffness; however, this finding has not been universally reported [36,88,89,90]. The effects of resistance exercise training on arterial stiffness were summaried in Table 3.

Previous research conducted by Yoon et al. investigated whether a short-term resistance exercise program increased arterial stiffness in healthy young men. Their result showed that HR (59.2 ± 9 vs. 80.4 ± 10.6 bpm), AIx (−6.3 vs. −2.6), and cfPWV were all significantly increased at 20 min post-exercise for the resistance exercise compared to the control group. Thus, in young healthy men, an acute resistance exercise program can increase arterial stiffness [88]. However, another study investigated the effects of an acute bout of free-weight/whole-body resistance exercise on cardiovascular modulations in resistance-trained individuals [36] and reported that the cfPWV and heart rate response increased after acute resistance exercise, suggesting that the whole-body free-weight style resistance training can increase arterial stiffness while decreasing vagal activity [36]. In contrast, another previous study examined the effect of leg push-up exercise on central and peripheral arterial stiffness in young adults performing their usual leg using PWV measurements [91]; the authors found that acute resistance exercise did not significantly change central arterial stiffness but appeared to reduce arterial stiffness in the exercising leg (preEx: 8.7 m/s; postEx-5 min: 7.5 m/s (*p* < 0.001, compared to preEx); postEx-25 min: 7.8 m/s (*p* < 0.05, compared to preEx)), but did not affect arterial stiffness in the non-exercised leg. Therefore, results from the above studies suggest that the effect of acute resistance exercise on arterial stiffness is equivocal; with some researchers reporting a decrease in arterial stiffness while others point to an increase. Further investigation is needed to determine the effects of long-term resistance training on arterial stiffness.

Based on our review of the literature, as well as findings from papers reported here, research involving resistance exercise programs have primarily focused on improving the musculoskeletal system rather than cardiovascular function. Contraction-induced muscle damage during intense resistance exercise has been linked to transient arterial stiffening due to both exercise-induced inflammation and increased muscle stiffness [92,93,94]. Unfortunately, most exercise protocols evaluating the effects of acute resistance exercise on vascular control in healthy individuals have been conducted using weight machines, [91,95], although there are some notable exceptions [96,97]. Thus, the cardiovascular response to resistance training with free weights may be different compared to performing resistance exercises using a weight machine. Free weight exercise may produce more muscle activation (i.e., more intense) compared to using a weight machine for resistance exercise. It is well established that the movements created with weight machines are limited to primary and stabilizing muscles [98,99]. Therefore, the resistance exercise involving greater muscle mass, when performing free weight resistance exercise, may lead to a transient increase in central arterial stiffness [90], which could be mediated by increased sympathetic activation after exercise [100]. Due to mechanical compression of blood vessels, an intense exercise pressor reflex, and execution of the Valsalva maneuver, extreme muscle hypertrophy can result in brief, intermittent increases in blood pressure, reaching up to four-fold resting values [1]. Taken together, the transient increase in inflammation and sympathetic activation may contribute to the temporary elevation in arterial stiffness following an acute bout of resistance exercise. Although, the specific type of resistance exercise (e.g., weight machine or free-weight exercise) must be considered when evaluating cardiovascular responses. Overall, acute resistance was demonstrated to have an antagonistic effect on arterial stiffness, with overall pulse wave velocity and augmentation index increases, possibly due to cardiovascular and non-cardiovascular factors.

If acute resistance exercise can produce negative impacts on arterial compliance, it would be interesting to know whether there are available interventions that might attenuate such acute perturbations. A recent study shows when performing resistance exercise with whole-body vibration (WBV) appears to cause low levels of cardiovascular stress and a reduction in systemic arterial stiffness reflected by measuring AIx75 (%). Figueroa et al. investigated the aortic hemodynamic and arterial responses after an acute bout of static squat exercise (commonly used movement in WBV training) with and without WBV [89]. Based on this study, AIx was elevated throughout the recovery after no-WBV while decreasing at 15 and 30 min after WBV exercise. baPWV was reduced at 5 min after both trials but returned to baseline at 15 min after no-WBV training (*p* < 0.01). Interestingly, there were no significant changes in cfPWV and baPWV after both tests [89]. The results suggest that WBV might be beneficial to attenuate the transient increase in arterial stiffness after acute resistance exercise, whereas the underlying mechanism for this benefit remains unknown.

Although there are controversial findings in acute resistance exercise on arterial stiffness, many lines of evidence still reveal that chronic resistance training exhibits clear benefits in improving arterial stiffness in varied populations [74,101,102]. Pedralli et al. (2020) demonstrated that 8 weeks of resistance training (RT: 6 resistance exercises, 4 × 12 reps, 60% 1 RM) improved FMD by 4.0% in individuals with pre-hypertension or hypertension [72], suggesting resistance exercise is capable of ameliorating endothelial functions in hypertensive populations [72]. In addition, 12 weeks of moderate-intensity resistance training (60% of 1 RM, 2 days/week) significantly increased maximal strength but did not impair both central and peripheral arterial compliance (measured by cfPWV and faPWV) in middle-aged women, suggesting that moderate-intensity resistance training did not increase arterial stiffness in this population [103]. However, another study by Turri-Silva and colleagues assessed the effects of 12 weeks of progressive high circuit resistance training (CRT; 3 sessions/week), on endothelial function and cardiopulmonary function in patients with heart failure (diagnosed New York Heart Association classification I and II) and reported no beneficial effect on vascular endothelial function [104]. These results further point out the possibility that the perturbations of resistance training on cardiovascular health could be different depending on populations, training intensity, and training frequency.

Another cellular and molecular mechanism for resistance exercise-improved arterial stiffness is that exercise ameliorates endothelial functions through endothelial progenitor cell (EPC) mobilization. Several lines of evidence revealed a clear correlation between circulating EPC numbers and vascular endothelial function, and damaged endothelial cell layers can be repaired by EPCs released from the bone marrow into the bloodstream to maintain the function and integrity of the vascular endothelium [101,102,105]. Ribeiro and colleagues investigated the effect of single resistance exercise of different intensities (60%, 70%, 80% 1RM; three sets of 12 repetitions of four large muscle group movements) on the mobilization of circulating EPCs [106]. The authors reported that circulating levels of EPCs and vascular endothelial growth factor (VEGF), hypoxia-inducible factor 1-alpha (HIF-1α), and erythropoietin (EPO) were significantly increased after exercise, and the increase in EPCs was greatest at 80% 1 RM exercise intensity [106], suggesting that resistance exercise promotes mobilization of EPCs in a dose–response relationship and possibly mediated through the above angiogenic factors responses (VEGF, HIF-1, and EPO).

Finally, we here have to note that combining strength resistance training with normobaric hypoxia or local blood flow restriction (BFR) has been reported to produce better and greater adaptations and beneficial physiological changes in muscle tissue, resulting in favorable phenotypic changes in skeletal muscle hypertrophy [107,108,109,110]. Also, previous research suggests that a wide range of movements during resistance training stimulates muscular hypertrophy; increasing muscle activity combined with extended time under tension could positively mediate intracellular anabolic signaling, promoting a more significant hypertrophic response [111]. However, there is still little known about whether such training modes consisting of limb-compression-induced blood flow restriction or low ambient oxygen on the changes in arterial stiffness-related biomarkers, and the acute and chronic impacts of these combined resistance training modes on cardiovascular functions warrant future investigations.

**Table 3:** Effects of resistance exercise training on arterial stiffness.

Authors	Subject	Research Design	Sample Size	Intervention	Intensity	Assessment Variable	Result/Outcomes
Yoon et al., 2010 [88]	Healthy non-smoking men Age: 20–29 years	The study involved a cross-over design in which the same subject was treated twice	13 subjects (Male: 13 and Female: 0)	Resistance exercise	Resistance exercises at 60% of 1 RM and sham control (seated rest) 15 repetitions, 2 sets	- cfPWV - HR - Aix Measured at: - Baseline (BL) - 20 min postEx - 40 min postEx	**HR (bpm)** - BL (59.2 ± 9) - 20 min (80.4 ± 10.6) ↑ - 40 min (73.5 ± 9.6) ↑ **AIx (%)** - BL (−6.3 (−13.4–0.8)) - 20 min (−2.6 (−10.3–5.1)) ↓ - 40 min (−5.4 (−12.6–1.9)) ↑ **cfPWV (m/s)** It was significantly elevated.
Figueroa et al., 2011 [89]	Healthy young men Age: 21 ± 4 years	This study used a cross-sectional design	15 subjects (Male: 15 and Female: 0)	Resistance exercise	10 rep of 1-min sets of static squats with/without WBV (40 Hz, 1 mm, 5.37 G), 10 sets	- AIx75 - baPWV - cfPWV Measured at: - Baseline - 5 min postEx - 15 min postEx - 30 min postEx	**Non-WBV Group** - AIx75 (%) (−6.9 ± 1.8 vs. 3.1 ± 2.8 vs. 2.6 ± 2.8 vs. −1.1 ± 2.5) (↑) - cfPWV (m/s) (8.7 ± 0.3 vs. 9.1 ± 0.4 vs. 8.9 ± 0.3 vs. 8.8 ± 0.2) (⟷) - baPWV (m/s) (12 ± 0.4 vs. 12 ± 0.3 vs. 12 ± 0.3 vs. 12.2 ± 0.4) (⟷) **WBV Group** - AIx75 (−6.1 ± 1.7 vs. −3.3 ± 3.0 vs. −9.1 ± 2.3 vs. −9.6 ± 2.1) (↓) - cfPWV (8.8 ± 0.3 vs. 9.1 ± 0.4 vs. 8.9 ± 0.3 vs. 8.5 ± 0.3) (⟷) - baPWV (12 ± 0.4 vs. 11.9 ± 0.3 vs. 11.8 ± 0.3 vs. 11.8 ± 0.3) (⟷)
Kingsley et al., 2016 [36]	Healthy young adults Age: 23 ± 3 years	This study used a cross-over study design	16 subjects (Male: 11 and Female: 5)	Whole Body Resistance exercise	3 sets of 10 repetitions at 75% 1 RM free-weight exercise (squat, deadlift, and bench press)	- cfPWV Measured at: - At rest - 0 min postEx	- **cfPWV (m/s)** CON (5.3 vs. 5.4) (⟷) RE (5.3 vs. 5.8) (↑)
Okamoto et al., 2014 [112]	Healthy young adults Age: 26 ± 5 years	This study used a randomized controlled crossover design	10 subjects (Male: 7 and Female: 3)	Resistance exercise	LRE (40% of 1 repetition maximum) and CON (seated rest in the exercise room), 3 sets until exhaustion	- Arterial compliance index - Carotid β-stiffness index Measured at: - Baseline (BL) - 30 min postEx - 60 min postEx	- **Arterial compliance (mm2/mmHg) [*p* < 0.05]** CON (0.13 vs. 0.12 vs. 0.12) (↑) LRE (0.13 vs. 0.17 vs. 0.17) (⟷) - **Carotid β-stiffness index (U) [*p* < 0.05]** CON (1.9 vs. 2.0 vs. 2.0) (⟷) LRE (2.0 vs. 1.5 vs. 1.3) (↓)

Abbreviations: AIx, Augmentation index; baPWV, brachial–ankle pulse wave velocity; BP, blood pressure; CAVI, cardio–ankle vascular index; CAE, continuous moderate-intensity aerobic exercise; cfPWV, carotid–femoral pulse wave velocity; CON, control; cSBP, central systolic blood pressure; faPWV, femoral–ankle pulse wave velocity; fdPWV, femoral dorsalis pedis pulse wave velocity; FMD, flow-mediated dilation; HIIT, high-intensity interval training; hIAE, high-intensity interval aerobic exercise; HIIE, high-intensity interval exercise; HR, heart rate; HRmax, heart rate maximum; IMT, intima media thickness; LRE, low-intensity resistance exercise; MCT, moderate continuous training; MICE, moderate-intensity continuous exercise; PWV, pulse wave velocity; SEVR, subendocardial viability ratio; T2D, type 2 diabetes; WBV, whole-body vibration; ⟷, no change; ↑, increase; ↓, decrease.

## 6. Effects of Interval Training on Arterial Stiffness

Obesity and metabolic disorders have been associated with increased arterial stiffness and the risk of CVD [42]. Although aerobic exercise has been shown to improve arterial stiffness, most recently HIIT has been shown to improve cardiorespiratory fitness and endothelial function. Regular aerobic exercise has been recognized as an effective preventive strategy to reduce central arterial stiffness [65]; in addition, both moderate continuous exercise (MCE; 35% HRR) and interval exercise (IE; 35% HRR) may temporarily improve the degree of arterial stiffness in humans. Further, both moderate continuous exercise (MCE; 35% HRR) and interval exercise (IE; 35% HRR) can temporarily improve the degree of arterial stiffness in humans, despite there being no difference in exercise intensity and duration, intermittent exercise patterns can further prolong the duration of improvement in arterial stiffness [33]. Knowing that both moderate and vigorous aerobic exercise is recommended for health maintenance, Hortman et al. took things a step further and evaluated the effects of HIIT. Thus, HIIT treadmill running (4 × 4 min at 85–95% of HRmax) may be an effective alternative to moderate-intensity continuous training (41 min at 65–75% of HRmax) for cardiometabolic disease prevention [113]. The findings of this study suggest that HIIT is safe and may have cardiac benefits by inducing transient peripheral vascular enhancements following just a single bout of exercise. The effects of interval exercise on arterial stiffness were summaried in Table 4.

A previous study investigated whether HIIT would improve cardiovascular outcomes in people with type 2 diabetes [114]. In people with T2D, HIIT reduced femoral IMT (fIMT; 0.84 ± 0.21 vs. 0.81 ± 0.16 mm; *p* = 0.03), cfPWV (10.1 ± 3.2 vs. 8.6 ± 1.8 m/s; *p* < 0.01), and resting heart rate (70.4 ± 10.8 vs. 67.8 ± 8.6 bpm; *p* = 0.01), suggesting that HIIT exhibited clear benefits on ameliorating arterial stiffness in diabetic populations [114]. A recent study by Agjaei Bahmanbeglou et al. compared the effects of two different HIIT protocols on arterial stiffness, lipid profiles, and inflammatory markers in hypertensive patients [115]. Of which, in patients with stage 1 hypertension, performing HIIT with intensity of 75–80% VO_2peak_ on a treadmill, improved systolic blood pressure and inflammatory markers regardless of HIIT intensity and duration, although improvements in PWV proved to be intensity-dependent [115]. Therefore, physical activity and arterial stiffness had an inverse relationship in that the more physically active one becomes the less likely they were to have increased arterial stiffness [116]. HIIT and moderate-intensity continuous training (MICT) produce comparable acute improvements in peripheral insulin sensitivity the day after exercise, as well as comparable long-term metabolic adaptations in skeletal muscle [117]. Also, HIIT exercise produced meaningful improvements in body composition, heart rate, blood pressure, and blood lipid metabolism. It was reported to affect the pulse wave reflection via increased blood flow and shear stress, resulting in reduced arterial stiffness. This suggests that HIIT may effectively decrease the probability of arterial stiffness while also protecting cardiovascular function.

On the other hand, several recent studies have investigated the differences in the effects of continuous aerobic/endurance exercise and other exercise training types such as resistance training [118] and HIIT [119] on arterial stiffness. Beneficial effects of high- and moderate-intensity resistance training on arterial stiffness and NO production were not observed in these intervention studies [120]. In a study by Cock et al. (2013), using Wingate-tested sprinting high-intensity exercise as the primary exercise for HIIT (30 s of sprinting + 4.5 min of 30 W low-intensity recovery for 4–6 sets; total exercise time 15.5–25.5 min), HIIT was found to significantly increase vascular eNOS expression in untrained healthy young men [121], suggesting that vascular shear stress induced by a single bout of HIIT may activate vascular endothelial eNOS and subsequent NO production. Although the effects on arterial stiffness may differ among distinct types of exercise, the molecular mechanisms underlying the differences in training effects remain unclear. If varying exercise programs induce different changes in arterial NO production, these findings may contribute to a reduction in exercise-induced arterial stiffness. A recent study by Hasegawa and colleagues comparing different training modes on changes in cardiovascular health status revealed a significant negative correlation between aortic PWV and the degree of endothelial eNOS phosphorylation in the aortic vasculature (r = −0.38, *p* < 0.05), these results suggest that there may be a causal relationship between increased arterial NO production and decreased arterial stiffness as a result of exercise [69], and may also be one of the possible molecular physiological mechanisms by which HIIT promotes increased arterial vascular compliance.

The effect of HIIT on arterial stiffness is similar to or greater than continuous aerobic/endurance exercise training [121,122,123]. Ramírez-Vélez and colleagues compared the cardiovascular benefits of moderate continuous training (MCT; 60–75% HRR for 35 min; 3 days/week) and HIIT (4 × 4 min at 85–95% HRR/4 × 4 min at 75–85% HRR; 3 days/week) in physically inactive adults, and the authors demonstrated that 12 weeks of HIIT is more effective in improving cardiovascular health in terms of improving FMD (MCT: −1.0% vs. HIIT: +1.8%) and decreasing PWV (MCT: +0.1 m·s^−1^ vs. HIIT: −0.4 m·s^−1^) compared to MCT in this population [124]. Additionally, a recent study by da Silva et al. (2020) reported that 12 weeks of HIIT (15 min) combined with physical activity (30 min/session; 3 sessions/week) significantly improved their physical fitness, body composition, and endothelial function (improving FMD by 4.5% above baseline) in obese adolescents, suggesting that HIIT training can prevent endothelial damage that precedes atherosclerosis development [125]. On the other hand, even HIIT with shorter exercise durations may lead to an effective and meaningful reduction in arterial stiffness [69]. A more recent study compared high-intensity interval exercise (HIIE) and moderate-intensity continuous exercise (MICE) with a control group (CON), and the results revealed a significant group x time effect for changes in central systolic blood pressure (F = 3.20, *p* = 0.01) with a transient reduction for the HIIE group but not the MICE or CON groups [32]. Among the various HIITs, the shorter duration exhaustive HIIT (4 min total duration) consisting of six or seven sets of 20 s exercises interspersed with 10 s rest interval periods significantly increased aerobic capacity over 6 weeks and was shown to be comparable to aerobic capacity induced by conventional aerobic/endurance exercise, although the total exercise volume was significantly less than that of long continuous endurance training [126].

Another previous study examined the effects of HIIT and moderate continuous training (MCT) on arterial pulse wave reflection and found that AIx@75 increased after both types of acute exercise but was higher after HIIT at t5 (*p* < 0.001), t20 (*p* < 0.001), and t35 (*p* = 0.009) compared to MCT [127]. Nevertheless, the impacts of HIIT on vascular health benefits might be varied in different populations. For example, in patients with heart failure (mean age 56 years; heart failure classification New York Heart Association classification I and II), 12 weeks of HIIT significantly improved cardiopulmonary fitness but not endothelial function, suggesting that the effect of HIIT on endothelial function may be less pronounced in patients with heart failure [104]. Taken together, HIIT may reduce arterial stiffness by effectively promoting NO bioavailability in central arteries, even during relatively short exercise durations, and the benefit is comparable to that of long-duration continuous aerobic/endurance exercise [69]; however, the benefits of HIIT could be varied in the populations with severe vascular structure impairments (i.e., heart failure).

Based on the available evidence, the main physiological mechanism underlying the positive effect of HIIT exercise on modulating endothelial function and arterial stiffness involves the upregulation of eNOS activity. However, compared to moderate continuous exercise, the greater effects of HIIT on promoting endothelial function may be since repetitive bouts of high-intensity exercise result in sustaining the endothelial function-promoting effects to exercise stimulation for a longer period, which in turn achieves a cumulative effect of exercise intervention in reducing arterial stiffness. Furthermore, differences in the reduction in arterial stiffness induced by different exercise regimens may be due to differences in the ability of exercise to modulate blood flow in working muscle vessels, which in turn promotes greater shear stress-induced NO bioavailability [128] and induces favorable endothelial adaptations [129]. However, studies directly focusing on the effects of HIIT on endothelial cellular senescence and endothelial function remain rare, and future investigations should consider assessing these possible factors mentioned above while exploring differential responses in different populations to fill their population-specific gaps in the literature of interest.

**Table 4:** Effect of interval exercise on arterial stiffness.

Authors	Subject	Research Design	Sample Size	Intervention	Intensity	Assessment Variable	Result/Outcomes
Hortmann et al., 2020 [113]	Young obese women Age: 18–39 years old	This study used a cross-over study design	15 subjects (Male: 0 and Female: 15)	HIIT	HIIT (4 × 4 min at 85–95% of HRmax), MICT (41 min at 65–75% of HRmax), and control	- cfPWV - AIx - AIx@75 Measured at: - Baseline (BL) - 1 min postEx - 30 min postEx - 60 min postEx	- **HIIT Group** AIx (%) [*p* < 0.001] (15 vs. 8 vs. 2 vs. (−1)) (↓) cfPWV (m/s) [*p* = 0.811] (6.5 vs. 6.4 vs. 6.5 vs. 6.5) (⟷) AIx@75 (%) [*p* = 0.049] (16 vs. 17 vs. 13 vs. 3) (↓) - **MICT Group** AIx (%) [*p* < 0.001] (15 vs. 14 vs. 9 vs. 8.5) (↓) cfPWV (m/s) [*p* = 0.811] (6.5 vs. 6.5 vs. 6.4 vs. 6.5) (⟷) AIx@75 (%) [*p* = 0.049] (18 vs. 17 vs. 10 vs. 9) (↓) - **Control Group** AIx (%) [*p* < 0.001] (20 vs. 16 vs. 15 vs. 16) (⟷) cfPWV (m/s) [*p* = 0.811] (6.4 vs. 6.4 vs. 6.3 vs. 6.2) (⟷) AIx@75 (%) [*p* = 0.049] (18 vs. 15 vs. 13 vs. 14) (⟷)
Francois et al., 2017 [114]	T2D adults patients VO_2peak_: 17.9 mL/min/kg Age: 57.6 ± 8.6 years	This study used a double-blind controlled trial	53 subjects (Male: 19 and Female: 34)	HIIT	Cardio and resistance-based HIIT (4–10 × 1 min at 90% HRmax)	Central and peripheral PWV Measured at: - 20 min preEx - 30 s postEx	HIIT reduces femoral IMT, arterial stiffness, and resting heart rate in individuals with T2D. - **HIIT femoral IMT [*p* = 0.03]** Pre: 0.84 ± 0.21 mm Post: 0.81 ± 0.16 mm (↓) - **cfPWV [*p* < 0.01]** Pre: 10.1 ± 3.2 m/s Post: 8.6 ± 1.8 m/s (↓) - **Resting HR [*p* = 0.01]** Pre: 70.4 ± 10.8 bpm Post: 67.8 ± 8.6 bpm (↓)
Hanssen et al., 2015 [127]	Healthy young men VO_2peak_: 4.2 ± 0.5 mL/min/kg Age: 18–35 years	This study used a randomized cross-over design	21 subjects (Male: 21 and Female: 0)	HIIT	HIIT (4 × 4 min interval training at 90–95% HRmax) and MCT (80% HR (±5 heartbeats))	- Aix - AIx75 - HR Measured at: - Baseline (BL) - 5 min postEx - 20 min postEx - 35 min postEx - 50 min postEx	- **AIx (%) [*p* = 0.024]** HIIT vs. MCT 0 min (−2 ± 8 vs. −2.6 ± 8) [*p* = 0.825] 5 min (−1.3 ± 9 vs. −3.7 ± 8) [*p* = 0.195] 20 min (−4 ± 8 vs. −2.7 ± 8) [*p* = 0.491] 35 min (−6 ± 8 vs. −2.2 ± 8) [*p* = 0.045] (↓) 50 min (−6.9 ± 8 vs. −1.9 ± 8) [*p* = 0.008] (↓) - **AIx75 (%) [*p* < 0.001]** HIIT vs. MCT 0 min (−10.8 ± 9 vs. −11.9 ± 8) [*p* = 0.663] 5 min (8.3 ± 9 vs. −4.4 ± 8) [*p* < 0.001] 20 min (1.4 ± 9 vs. −7.9 ± 8) [*p* < 0.001] 35 min (−4.1 ± 9 vs. −9.5 ± 8) [*p* = 0.009] (↓) 50 min (−7.4 ± 9 vs. −10.2 ± 8) [*p* = 0.206] (↓)

Abbreviations: AIx, Augmentation index; AIx75: Augmentation index adjusted for 75 beats per minute; baPWV, brachial–ankle pulse wave velocity; BP, blood pressure; CAVI, cardio–ankle vascular index; CAE, continuous moderate-intensity aerobic exercise; cfPWV, carotid–femoral pulse wave velocity; CON, control; cSBP, central systolic blood pressure; faPWV, femoral–ankle pulse wave velocity; fdPWV, femoral dorsalis pedis pulse wave velocity; FMD, flow-mediated dilation; HIIT, high-intensity interval training; hIAE, high-intensity interval aerobic exercise; HIIE, high-intensity interval exercise; HR, heart rate; HRmax, heart rate maximum; IMT, intima media thickness; LRE, low-intensity resistance exercise; MCT, moderate continuous training; MICE, moderate-intensity continuous exercise; PWV, pulse wave velocity; SEVR, subendocardial viability ratio; T2D, type 2 diabetes; WBV, whole-body vibration; ⟷, no change; ↓, decrease.

## 7. Conclusive Remarks and Suggestions for Future Research

**Figure 1:**
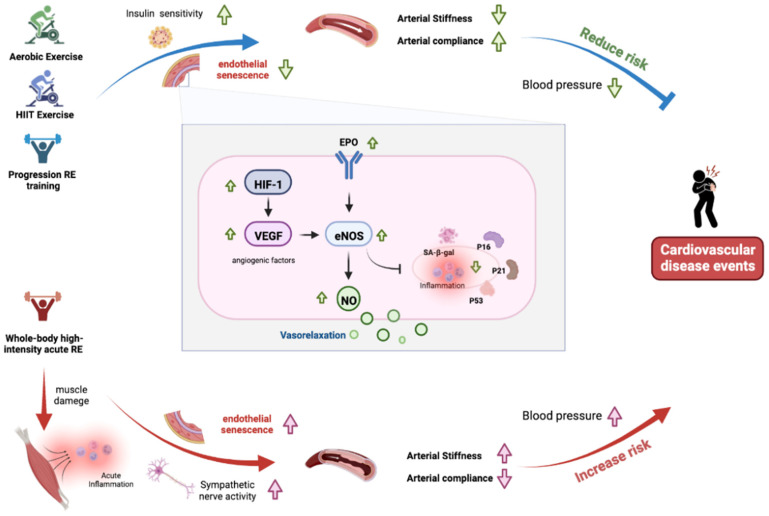
Arterial stiffness is an independent predictor of cardiovascular events. Different modes of exercise may have different effects on arterial stiffness. Aerobic, HIIT, and Progression RE-induced physiological and cellular levels of endothelial to promote angiogenic factors responses (VEGF, HIF-1, and EPO) and improve nitric oxide (NO) bioavailability by altering endothelial senescence markers (p53, p21, p16, and SA-β-gal), which attenuate the deleterious changes in blood pressure and vascular functions, including the increase in arterial compliance and the decrease in arterial stiffness. However, whole-body high-intensity acute RE-induced muscle damage, which leads to the transient increase in inflammation, endothelial senescence, and sympathetic activation may contribute to the temporary elevation in blood pressure and arterial stiffness. RE: resistance exercise; VEGF: vascular endothelial growth factor; HIF-1α: hypoxia-inducible factor 1-alpha EPO: erythropoietin; eNOS: endothelial nitric oxide synthase; SA-β-gal: senescence-associated-β-galactosidase.

Arterial stiffness and EC senescence stand out as the main cardiovascular events. Some of the cellular biomarkers associated with cellular senescence have also been identified to be associated with decreased vascular EC function. This impact is related to increased expression of p53, p21, p16, and SA-β-gal activity in the arterial tissue with oxidative stress-mediated inhibition of NO-dependent vascular endothelial function. In this review, the recent scientific literature has been carefully discussed to demonstrate that aerobic, HIIT, and Progression RE-induced arterial compliance may reduce arterial stiffness by effectively promoting NO bioavailability and angiogenic factor responses (VEGF, HIF-1, and EPO), as well as reducing endothelial senescence. However, the transient increase in inflammation and sympathetic activation may contribute to the temporary elevation in arterial stiffness following a whole-body high-intensity acute resistance exercise (Figure 1). If acute resistance exercise can produce negative impacts on arterial compliance, it would be interesting to know whether there are available interventions that might attenuate such acute perturbations. Training modes consisting of limb-compression-induced blood flow restriction or low ambient oxygen on the changes in arterial stiffness-related biomarkers, and the acute and chronic impacts of these combined resistance training modes on cardiovascular functions and related underlying mechanisms remain unclear and need further investigation.

Based on the existing evidence, the effects of different exercise patterns involving acute exercise challenges or chronic training adaptations on arterial stiffness are complicated. Moreover, changes in arterial compliance vary in duration, intensity, and type of exercise. Exercise-induced changes in arterial stiffness may involve different physiological regulations, muscle damage/inflammatory responses, and molecular mechanisms controlling endothelial senescence, and subsequently exert multiple benefits. This review focuses on the effects of different types of exercise from the perspective of endothelial cell senescence and arterial stiffness, and therefore may not fully cover the entire systemic pathological changes and the possible effects of neurohormonal regulatory mechanisms involved. We suggest that a comprehensive discussion and review of these aspects could be conducted in the future. However, given the benefits of regular exercise on the cardiovascular system and the preventive effects of future cardiovascular events, it is now more important than ever that we maintain the momentum of exercise-related research regarding cardiovascular aspects to better understand the involved underlying mechanisms to improve overall health and wellness.

## Data Availability

Not applicable.

## References

[1] Pierce D.R., Doma K., Leicht A.S. (2018). Acute Effects of Exercise Mode on Arterial Stiffness and Wave Reflection in Healthy Young Adults: A Systematic Review and Meta-Analysis. Front. Physiol..

[2] Said M.A., Eppinga R.N., Lipsic E., Verweij N., van der Harst P. (2018). Relationship of Arterial Stiffness Index and Pulse Pressure with Cardiovascular Disease and Mortality. J. Am. Heart Assoc..

[3] Wu M., Shu Y., Wang L., Song L., Chen S., Liu Y., Bi J., Li D., Yang Y., Hu Y. (2021). Visit-to-visit variability in the measurements of metabolic syndrome components and the risk of all-cause mortality, cardiovascular disease, and arterial stiffness. Nutr. Metab. Cardiovasc. Dis. NMCD.

[4] Zhang Y., Agnoletti D., Xu Y., Wang J.G., Blacher J., Safar M.E. (2014). Carotid-femoral pulse wave velocity in the elderly. J. Hypertens..

[5] Bonarjee V.V.S. (2018). Arterial Stiffness: A Prognostic Marker in Coronary Heart Disease. Available Methods and Clinical Application. Front. Cardiovasc. Med..

[6] Van Bortel L.M., Laurent S., Boutouyrie P., Chowienczyk P., Cruickshank J.K., De Backer T., Filipovsky J., Huybrechts S., Mattace-Raso F.U., Protogerou A.D. (2012). Expert consensus document on the measurement of aortic stiffness in daily practice using carotid-femoral pulse wave velocity. J. Hypertens..

[7] Chirinos J.A., Kips J.G., Jacobs D.R., Brumback L., Duprez D.A., Kronmal R., Bluemke D.A., Townsend R.R., Vermeersch S., Segers P. (2012). Arterial wave reflections and incident cardiovascular events and heart failure: MESA (Multiethnic Study of Atherosclerosis). J. Am. Coll. Cardiol..

[8] Safar M.E., Balkau B., Lange C., Protogerou A.D., Czernichow S., Blacher J., Levy B.I., Smulyan H. (2013). Hypertension and vascular dynamics in men and women with metabolic syndrome. J. Am. Coll. Cardiol..

[9] Safar M.E., Thomas F., Blacher J., Nzietchueng R., Bureau J.M., Pannier B., Benetos A. (2006). Metabolic syndrome and age-related progression of aortic stiffness. J. Am. Coll. Cardiol..

[10] Meyer M.L., Tanaka H., Palta P., Cheng S., Gouskova N., Aguilar D., Heiss G. (2016). Correlates of Segmental Pulse Wave Velocity in Older Adults: The Atherosclerosis Risk in Communities (ARIC) Study. Am. J. Hypertens..

[11] Perissiou M., Bailey T.G., Windsor M., Nam M.C.Y., Greaves K., Leicht A.S., Golledge J., Askew C.D. (2018). Effects of exercise intensity and cardiorespiratory fitness on the acute response of arterial stiffness to exercise in older adults. Eur. J. Appl. Physiol..

[12] Tune J.D., Goodwill A.G., Sassoon D.J., Mather K.J. (2017). Cardiovascular consequences of metabolic syndrome. Transl. Res..

[13] Pannier B., Thomas F., Eschwège E., Bean K., Benetos A., Leocmach Y., Danchin N., Guize L. (2006). Cardiovascular risk markers associated with the metabolic syndrome in a large French population: The “SYMFONIE” study. Diabetes. Metab..

[14] Akbulut G., Köksal E., Bilici S., Acar Tek N., Yildiran H., Karadag M.G., Sanlier N. (2011). Metabolic syndrome (MS) in elderly: A cross sectional survey. Arch. Gerontol. Geriatr..

[15] Urbina E.M., Gao Z., Khoury P.R., Martin L.J., Dolan L.M. (2012). Insulin resistance and arterial stiffness in healthy adolescents and young adults. Diabetologia.

[16] Webb D.R., Khunti K., Silverman R., Gray L.J., Srinivasan B., Lacy P.S., Williams B., Davies M.J. (2010). Impact of metabolic indices on central artery stiffness: Independent association of insulin resistance and glucose with aortic pulse wave velocity. Diabetologia.

[17] Won K.B., Park G.M., Lee S.E., Cho I.J., Kim H.C., Lee B.K., Chang H.J. (2018). Relationship of insulin resistance estimated by triglyceride glucose index to arterial stiffness. Lipids Health Dis..

[18] Humphrey J.D. (2021). Mechanisms of Vascular Remodeling in Hypertension. Am. J. Hypertens..

[19] Lopes-Vicente W.R.P., Rodrigues S., Cepeda F.X., Jordão C.P., Costa-Hong V., Dutra-Marques A.C.B., Carvalho J.C., Alves M., Bortolotto L.A., Trombetta I.C. (2017). Arterial stiffness and its association with clustering of metabolic syndrome risk factors. Diabetol. Metab. Syndr..

[20] Vatner S.F., Zhang J., Vyzas C., Mishra K., Graham R.M., Vatner D.E. (2021). Vascular Stiffness in Aging and Disease. Front. Physiol..

[21] Safar M.E., Asmar R., Benetos A., Blacher J., Boutouyrie P., Lacolley P., Laurent S., London G., Pannier B., Protogerou A. (2018). Interaction Between Hypertension and Arterial Stiffness. Hypertension.

[22] Shephard R.J., Balady G.J. (1999). Exercise as cardiovascular therapy. Circulation.

[23] Ryan A.S. (2000). Insulin resistance with aging. Sport. Med..

[24] Carracedo J., Ramírez-Carracedo R., Alique M., Ramírez-Chamond R. (2018). Endothelial cell senescence in the pathogenesis of endothelial dysfunction. Endothel Dysfunct Old Concepts New Challenges.

[25] Lloyd-Jones D.M. (2010). Cardiovascular risk prediction: Basic concepts, current status, and future directions. Circulation.

[26] Benjamin E.J., Blaha M.J., Chiuve S.E., Cushman M., Das S.R., Deo R., de Ferranti S.D., Floyd J., Fornage M., Gillespie C. (2017). Heart Disease and Stroke Statistics-2017 Update: A Report From the American Heart Association. Circulation.

[27] Seals D.R., Jablonski K.L., Donato A.J. (2011). Aging and vascular endothelial function in humans. Clin. Sci..

[28] Jia G., Aroor A.R., DeMarco V.G., Martinez-Lemus L.A., Meininger G.A., Sowers J.R. (2015). Vascular stiffness in insulin resistance and obesity. Front. Physiol..

[29] Gill J.M., Al-Mamari A., Ferrell W.R., Cleland S.J., Packard C.J., Sattar N., Petrie J.R., Caslake M.J. (2004). Effects of prior moderate exercise on postprandial metabolism and vascular function in lean and centrally obese men. J. Am. Coll. Cardiol..

[30] Gruber H.J., Mayer C., Mangge H., Fauler G., Grandits N., Wilders-Truschnig M. (2008). Obesity reduces the bioavailability of nitric oxide in juveniles. Int. J. Obes..

[31] Kim F., Pham M., Maloney E., Rizzo N.O., Morton G.J., Wisse B.E., Kirk E.A., Chait A., Schwartz M.W. (2008). Vascular inflammation, insulin resistance, and reduced nitric oxide production precede the onset of peripheral insulin resistance. Arter. Thromb. Vasc. Biol..

[32] Way K.L., Lee A.S., Twigg S.M., Johnson N.A. (2021). The effect of acute aerobic exercise on central arterial stiffness, wave reflections, and hemodynamics in adults with diabetes: A randomized cross-over design. J. Sport Health Sci..

[33] Wang H., Zhang T., Zhu W., Wu H., Yan S. (2014). Acute effects of continuous and interval low-intensity exercise on arterial stiffness in healthy young men. Eur. J. Appl. Physiol..

[34] Siasos G., Athanasiou D., Terzis G., Stasinaki A., Oikonomou E., Tsitkanou S., Kolokytha T., Spengos K., Papavassiliou A.G., Tousoulis D. (2016). Acute effects of different types of aerobic exercise on endothelial function and arterial stiffness. Eur. J. Prev. Cardiol..

[35] Doonan R.J., Mutter A., Egiziano G., Gomez Y.H., Daskalopoulou S.S. (2013). Differences in arterial stiffness at rest and after acute exercise between young men and women. Hypertens. Res..

[36] Kingsley J.D., Mayo X., Tai Y.L., Fennell C. (2016). Arterial Stiffness and Autonomic Modulation After Free-Weight Resistance Exercises in Resistance Trained Individuals. J. Strength Cond. Res..

[37] Scuteri A., Najjar S.S., Orru M., Usala G., Piras M.G., Ferrucci L., Cao A., Schlessinger D., Uda M., Lakatta E.G. (2010). The central arterial burden of the metabolic syndrome is similar in men and women: The SardiNIA Study. Eur. Heart J..

[38] Topouchian J., Labat C., Gautier S., Back M., Achimastos A., Blacher J., Cwynar M., de la Sierra A., Pall D., Fantin F. (2018). Effects of metabolic syndrome on arterial function in different age groups: The Advanced Approach to Arterial Stiffness study. J. Hypertens..

[39] Blacher J., Guerin A.P., Pannier B., Marchais S.J., Safar M.E., London G.M. (1999). Impact of aortic stiffness on survival in end-stage renal disease. Circulation.

[40] Boutouyrie P., Tropeano A.I., Asmar R., Gautier I., Benetos A., Lacolley P., Laurent S. (2002). Aortic stiffness is an independent predictor of primary coronary events in hypertensive patients: A longitudinal study. Hypertension.

[41] Mattace-Raso F.U., van der Cammen T.J., Hofman A., van Popele N.M., Bos M.L., Schalekamp M.A., Asmar R., Reneman R.S., Hoeks A.P., Breteler M.M. (2006). Arterial stiffness and risk of coronary heart disease and stroke: The Rotterdam Study. Circulation.

[42] Cecelja M., Chowienczyk P. (2012). Role of arterial stiffness in cardiovascular disease. J. R. Soc. Med. Cardiovasc. Dis..

[43] Scuteri A., Cunha P.G., Agabiti Rosei E., Badariere J., Bekaert S., Cockcroft J.R., Cotter J., Cucca F., De Buyzere M.L., De Meyer T. (2014). Arterial stiffness and influences of the metabolic syndrome: A cross-countries study. Atherosclerosis.

[44] Henry R.M., Kostense P.J., Spijkerman A.M., Dekker J.M., Nijpels G., Heine R.J., Kamp O., Westerhof N., Bouter L.M., Stehouwer C.D. (2003). Arterial stiffness increases with deteriorating glucose tolerance status: The Hoorn Study. Circulation.

[45] Ho C.T., Lin C.C., Hsu H.S., Liu C.S., Davidson L.E., Li T.C., Li C.I., Lin W.Y. (2011). Arterial Stiffness is Strongly Associated with Insulin Resistance in Chinese—A Population-Based Study (Taichung Community Health Study, TCHS). J. Atheroscler. Thromb..

[46] Ryder J.R., Dengel D.R., Jacobs D.R., Sinaiko A.R., Kelly A.S., Steinberger J. (2016). Relations among Adiposity and Insulin Resistance with Flow-Mediated Dilation, Carotid Intima-Media Thickness, and Arterial Stiffness in Children. J. Pediatr..

[47] Nakagomi A., Sunami Y., Kawasaki Y., Fujisawa T., Kobayashi Y. (2020). Sex difference in the association between surrogate markers of insulin resistance and arterial stiffness. J. Diabetes Its Complicat..

[48] Hughes T.M., Althouse A.D., Niemczyk N.A., Hawkins M.S., Kuipers A.L., Tyrrell K.S. (2012). Effects of weight loss and insulin reduction on arterial stiffness in the SAVE trial. Cardiovasc. Diabetol..

[49] Fantin F., Comellato G., Rossi A.P., Grison E., Zoico E., Mazzali G., Zamboni M. (2017). Relationship between neck circumference, insulin resistance and arterial stiffness in overweight and obese subjects. Eur. J. Prev. Cardiol..

[50] Sengstock D.M., Vaitkevicius P.V., Supiano M.A. (2005). Arterial stiffness is related to insulin resistance in nondiabetic hypertensive older adults. J. Clin. Endocrinol. Metab..

[51] Matsushita H., Chang E., Glassford A.J., Cooke J.P., Chiu C.P., Tsao P.S. (2001). eNOS activity is reduced in senescent human endothelial cells: Preservation by hTERT immortalization. Circ. Res..

[52] Sato I., Morita I., Kaji K., Ikeda M., Nagao M., Murota S. (1993). Reduction of nitric oxide producing activity associated with in vitro aging in cultured human umbilical vein endothelial cell. Biochem. Biophys. Res. Commun..

[53] Yoon H.J., Cho S.W., Ahn B.W., Yang S.Y. (2010). Alterations in the activity and expression of endothelial NO synthase in aged human endothelial cells. Mech. Ageing Dev..

[54] Haendeler J., Hoffmann J., Diehl J.F., Vasa M., Spyridopoulos I., Zeiher A.M., Dimmeler S. (2004). Antioxidants inhibit nuclear export of telomerase reverse transcriptase and delay replicative senescence of endothelial cells. Circ. Res..

[55] Xin M.G., Zhang J., Block E.R., Patel J.M. (2003). Senescence-enhanced oxidative stress is associated with deficiency of mitochondrial cytochrome c oxidase in vascular endothelial cells. Mech. Ageing Dev..

[56] Donato A.J., Gano L.B., Eskurza I., Silver A.E., Gates P.E., Jablonski K., Seals D.R. (2009). Vascular endothelial dysfunction with aging: Endothelin-1 and endothelial nitric oxide synthase. Am. J. Physiol. Heart Circ. Physiol..

[57] Bhayadia R., Schmidt B.M., Melk A., Hömme M. (2016). Senescence-Induced Oxidative Stress Causes Endothelial Dysfunction. J. Gerontology. Ser. A Biol. Sci. Med. Sci..

[58] Werner C., Fürster T., Widmann T., Pöss J., Roggia C., Hanhoun M., Scharhag J., Büchner N., Meyer T., Kindermann W. (2009). Physical exercise prevents cellular senescence in circulating leukocytes and in the vessel wall. Circulation.

[59] Sato Y., Nagasaki M., Nakai N., Fushimi T. (2003). Physical exercise improves glucose metabolism in lifestyle-related diseases. Exp. Biol. Med..

[60] Sui X., LaMonte M.J., Laditka J.N., Hardin J.W., Chase N., Hooker S.P., Blair S.N. (2007). Cardiorespiratory fitness and adiposity as mortality predictors in older adults. J. Am. Med. Assoc..

[61] Powell K.E., Thompson P.D., Caspersen C.J., Kendrick J.S. (1987). Physical activity and the incidence of coronary heart disease. Annu. Rev. Public Health.

[62] Blair S.N., Kohl H.W., Paffenbarger R.S., Clark D.G., Cooper K.H., Gibbons L.W. (1989). Physical fitness and all-cause mortality: A prospective study of healthy men and women. J. Am. Med. Assoc..

[63] Vaitkevicius P.V., Fleg J.L., Engel J.H., O’Connor F.C., Wright J.G., Lakatta L.E., Yin F.C., Lakatta E.G. (1993). Effects of age and aerobic capacity on arterial stiffness in healthy adults. Circulation.

[64] Tanaka H., DeSouza C.A., Seals D.R. (1998). Absence of age-related increase in central arterial stiffness in physically active women. Arterioscler. Thromb. Vasc. Biol..

[65] Tanaka H., Dinenno F.A., Monahan K.D., Clevenger C.M., DeSouza C.A., Seals D.R. (2000). Aging, habitual exercise, and dynamic arterial compliance. Circulation.

[66] Cameron J.D., Dart A.M. (1994). Exercise training increases total systemic arterial compliance in humans. Am. J. Physiol.-Heart Circ. Physiol..

[67] Gando Y., Yamamoto K., Murakami H., Ohmori Y., Kawakami R., Sanada K., Higuchi M., Tabata I., Miyachi M. (2010). Longer time spent in light physical activity is associated with reduced arterial stiffness in older adults. Hypertension.

[68] Seals D.R., DeSouza C.A., Donato A.J., Tanaka H. (2008). Habitual exercise and arterial aging. J. Appl. Physiol..

[69] Hasegawa N., Fujie S., Horii N., Miyamoto-Mikami E., Tsuji K., Uchida M., Hamaoka T., Tabata I., Iemitsu M. (2018). Effects of Different Exercise Modes on Arterial Stiffness and Nitric Oxide Synthesis. Med. Sci. Sport. Exerc..

[70] Yu S., Yarnell J.W., Sweetnam P.M., Murray L. (2003). What level of physical activity protects against premature cardiovascular death? The Caerphilly study. Heart.

[71] Fransson E.I., Alfredsson L.S., de Faire U.H., Knutsson A., Westerholm P.J. (2003). Leisure time, occupational and household physical activity, and risk factors for cardiovascular disease in working men and women: The WOLF study. Scand. J. Public Health.

[72] Pedralli M.L., Marschner R.A., Kollet D.P., Neto S.G., Eibel B., Tanaka H., Lehnen A.M. (2020). Different exercise training modalities produce similar endothelial function improvements in individuals with prehypertension or hypertension: A randomized clinical trial Exercise, endothelium and blood pressure. Sci. Rep..

[73] Guimaraes G.V., Ciolac E.G., Carvalho V.O., D’Avila V.M., Bortolotto L.A., Bocchi E.A. (2010). Effects of continuous vs. interval exercise training on blood pressure and arterial stiffness in treated hypertension. Hypertens. Res..

[74] Asamoah S., Siegler J., Chang D., Scholey A., Yeung A., Cheema B.S. (2013). Effect of Aerobic Training on Cognitive Function and Arterial Stiffness in Sedentary Young Adults: A Pilot Randomized Controlled Trial. Physiol. J..

[75] Eskurza I., Monahan K.D., Robinson J.A., Seals D.R. (2004). Effect of acute and chronic ascorbic acid on flow-mediated dilatation with sedentary and physically active human ageing. J. Physiol..

[76] Rossman M.J., Kaplon R.E., Hill S.D., McNamara M.N., Santos-Parker J.R., Pierce G.L., Seals D.R., Donato A.J. (2017). Endothelial cell senescence with aging in healthy humans: Prevention by habitual exercise and relation to vascular endothelial function. Am. J. Physiol.-Heart Circ. Physiol..

[77] Schafer M.J., White T.A., Evans G., Tonne J.M., Verzosa G.C., Stout M.B., Mazula D.L., Palmer A.K., Baker D.J., Jensen M.D. (2016). Exercise Prevents Diet-Induced Cellular Senescence in Adipose Tissue. Diabetes.

[78] Thijssen D.H., Maiorana A.J., O’Driscoll G., Cable N.T., Hopman M.T., Green D.J. (2010). Impact of inactivity and exercise on the vasculature in humans. Eur. J. Appl. Physiol..

[79] Lee D.C., Pate R.R., Lavie C.J., Sui X., Church T.S., Blair S.N. (2014). Leisure-time running reduces all-cause and cardiovascular mortality risk. J. Am. Coll. Cardiol..

[80] Dawson E.A., Green D.J., Cable N.T., Thijssen D.H. (2013). Effects of acute exercise on flow-mediated dilatation in healthy humans. J. Appl. Physiol..

[81] Goto C., Higashi Y., Kimura M., Noma K., Hara K., Nakagawa K., Kawamura M., Chayama K., Yoshizumi M., Nara I. (2003). Effect of different intensities of exercise on endothelium-dependent vasodilation in humans: Role of endothelium-dependent nitric oxide and oxidative stress. Circulation.

[82] Ashor A.W., Lara J., Siervo M., Celis-Morales C., Mathers J.C. (2014). Effects of exercise modalities on arterial stiffness and wave reflection: A systematic review and meta-analysis of randomized controlled trials. PLoS ONE.

[83] Arce Esquivel A.A., Welsch M.A. (2007). High and low volume resistance training and vascular function. Int. J. Sport. Med..

[84] Okamoto T., Masuhara M., Ikuta K. (2011). Effect of low-intensity resistance training on arterial function. Eur. J. Appl. Physiol..

[85] Shiroma E.J., Cook N.R., Manson J.E., Moorthy M.V., Buring J.E., Rimm E.B., Lee I.M. (2017). Strength Training and the Risk of Type 2 Diabetes and Cardiovascular Disease. Med. Sci. Sport. Exerc..

[86] Chomistek A.K., Cook N.R., Flint A.J., Rimm E.B. (2012). Vigorous-intensity leisure-time physical activity and risk of major chronic disease in men. Med. Sci. Sport. Exerc..

[87] Tanasescu M., Leitzmann M.F., Rimm E.B., Willett W.C., Stampfer M.J., Hu F.B. (2002). Exercise type and intensity in relation to coronary heart disease in men. JAMA.

[88] Yoon E.S., Jung S.J., Cheun S.K., Oh Y.S., Kim S.H., Jae S.Y. (2010). Effects of Acute Resistance Exercise on Arterial Stiffness in Young Men. Korean Circ. J..

[89] Figueroa A., Vicil F., Sanchez-Gonzalez M.A. (2011). Acute exercise with whole-body vibration decreases wave reflection and leg arterial stiffness. Am. J. Cardiovasc. Dis..

[90] Failla M., Grappiolo A., Emanuelli G., Vitale G., Fraschini N., Bigoni M., Grieco N., Denti M., Giannattasio C., Mancia G. (1999). Sympathetic tone restrains arterial distensibility of healthy and atherosclerotic subjects. J. Hypertens..

[91] Heffernan K.S., Rossow L., Jae S.Y., Shokunbi H.G., Gibson E.M., Fernhall B. (2006). Effect of single-leg resistance exercise on regional arterial stiffness. Eur. J. Appl. Physiol..

[92] Barnes J.N., Trombold J.R., Dhindsa M., Lin H.-F., Tanaka H. (2010). Arterial stiffening following eccentric exercise-induced muscle damage. J. Appl. Physiol..

[93] Lin H.-F., Chou C.-C., Cheng H.-M., Tanaka H. (2017). Delayed onset vascular stiffening induced by eccentric resistance exercise and downhill running. Clin. J. Sport Med..

[94] Howell J., Chleboun G., Conatser R. (1993). Muscle stiffness, strength loss, swelling and soreness following exercise-induced injury in humans. J. Physiol..

[95] DeVan A.E., Anton M.M., Cook J.N., Neidre D.B., Cortez-Cooper M.Y., Tanaka H. (2005). Acute effects of resistance exercise on arterial compliance. J. Appl. Physiol..

[96] Lefferts W.K., Augustine J.A., Heffernan K.S. (2014). Effect of acute resistance exercise on carotid artery stiffness and cerebral blood flow pulsatility. Front. Physiol..

[97] Chen J.L., Yeh D.P., Lee J.P., Chen C.Y., Huang C.Y., Lee S.D., Chen C.C., Kuo T.B., Kao C.L., Kuo C.H. (2011). Parasympathetic nervous activity mirrors recovery status in weightlifting performance after training. J. Strength Cond. Res..

[98] Escamilla R.F., Fleisig G.S., Zheng N., Lander J.E., Barrentine S.W., Andrews J.R., Bergemann B.W., Moorman C.T. (2001). Effects of technique variations on knee biomechanics during the squat and leg press. Med. Sci. Sport. Exerc..

[99] Santana J.C., Vera-Garcia F.J., McGill S.M. (2007). A kinetic and electromyographic comparison of the standing cable press and bench press. J. Strength Cond. Res..

[100] Seals D.R. (1993). Influence of active muscle size on sympathetic nerve discharge during isometric contractions in humans. J. Appl. Physiol..

[101] Leone A.M., Valgimigli M., Giannico M.B., Zaccone V., Perfetti M., D’Amario D., Rebuzzi A.G., Crea F. (2009). From bone marrow to the arterial wall: The ongoing tale of endothelial progenitor cells. Eur. Heart J..

[102] Liao Y., Chen L., Zeng T., Li Y., Yu F., Hu L., Yue L. (2010). Number of circulating endothelial progenitor cells as a marker of vascular endothelial function for type 2 diabetes. Vasc. Med..

[103] Yoshizawa M., Maeda S., Miyaki A., Misono M., Saito Y., Tanabe K., Kuno S., Ajisaka R. (2009). Effect of 12 weeks of moderate-intensity resistance training on arterial stiffness: A randomised controlled trial in women aged 32–59 years. Br. J. Sport. Med..

[104] Turri-Silva N., Vale-Lira A., Verboven K., Quaglioti Durigan J.L., Hansen D., Cipriano G. (2021). High-intensity interval training versus progressive high-intensity circuit resistance training on endothelial function and cardiorespiratory fitness in heart failure: A preliminary randomized controlled trial. PLoS ONE.

[105] Urbich C., Dimmeler S. (2004). Endothelial progenitor cells: Characterization and role in vascular biology. Circ. Res..

[106] Ribeiro F., Ribeiro I.P., Gonçalves A.C., Alves A.J., Melo E., Fernandes R., Costa R., Sarmento-Ribeiro A.B., Duarte J.A., Carreira I.M. (2017). Effects of resistance exercise on endothelial progenitor cell mobilization in women. Sci. Rep..

[107] Fernández-Lázaro D., Díaz J., Caballero A., Córdova A. (2019). The training of strength-resistance in hypoxia: Effect on muscle hypertrophy. Biomed. Rev. Del. Inst. Nac. Salud.

[108] Centner C., Wiegel P., Gollhofer A., König D. (2019). Effects of Blood Flow Restriction Training on Muscular Strength and Hypertrophy in Older Individuals: A Systematic Review and Meta-Analysis. Sport. Med..

[109] Patterson S.D., Hughes L., Warmington S., Burr J., Scott B.R., Owens J., Abe T., Nielsen J.L., Libardi C.A., Laurentino G. (2019). Blood Flow Restriction Exercise: Considerations of Methodology, Application, and Safety. Front. Physiol..

[110] Pope Z.K., Willardson J.M., Schoenfeld B.J. (2013). Exercise and blood flow restriction. J. Strength Cond. Res..

[111] Wilk M., Zajac A., Tufano J.J. (2021). The Influence of Movement Tempo During Resistance Training on Muscular Strength and Hypertrophy Responses: A Review. Sport. Med..

[112] Okamoto T., Min S., Sakamaki-Sunaga M. (2014). Arterial compliance and stiffness following low-intensity resistance exercise. Eur. J. Appl. Physiol..

[113] Hortmann K., Boutouyrie P., Locatelli J.C., de Oliveira G.H., Simoes C.F., de Souza Mendes V.H., Reck H.B., Okawa R.T.P., Lopes W.A. (2021). Acute effects of high-intensity interval training and moderate-intensity continuous training on arterial stiffness in young obese women. Eur. J. Prev. Cardiol..

[114] Francois M.E., Pistawka K.J., Halperin F.A., Little J.P. (2018). Cardiovascular benefits of combined interval training and post-exercise nutrition in type 2 diabetes. J. Diabetes Its Complicat..

[115] Aghaei Bahmanbeglou N., Ebrahim K., Maleki M., Nikpajouh A., Ahmadizad S. (2019). Short-Duration High-Intensity Interval Exercise Training Is More Effective Than Long Duration for Blood Pressure and Arterial Stiffness But Not for Inflammatory Markers and Lipid Profiles in Patients With Stage 1 Hypertension. J. Cardiopulm. Rehabil. Prev..

[116] Endes S., Schaffner E., Caviezel S., Dratva J., Autenrieth C.S., Wanner M., Martin B., Stolz D., Pons M., Turk A. (2016). Physical activity is associated with lower arterial stiffness in older adults: Results of the SAPALDIA 3 Cohort Study. Eur. J. Epidemiol..

[117] Ryan B.J., Schleh M.W., Ahn C., Ludzki A.C., Gillen J.B., Varshney P., Van Pelt D.W., Pitchford L.M., Chenevert T.L., Gioscia-Ryan R.A. (2020). Moderate-Intensity Exercise and High-Intensity Interval Training Affect Insulin Sensitivity Similarly in Obese Adults. J. Clin. Endocrinol. Metab..

[118] Collier S.R., Kanaley J.A., Carhart R., Frechette V., Tobin M.M., Hall A.K., Luckenbaugh A.N., Fernhall B. (2008). Effect of 4 weeks of aerobic or resistance exercise training on arterial stiffness, blood flow and blood pressure in pre- and stage-1 hypertensives. J. Hum. Hypertens..

[119] Rakobowchuk M., Tanguay S., Burgomaster K.A., Howarth K.R., Gibala M.J., MacDonald M.J. (2008). Sprint interval and traditional endurance training induce similar improvements in peripheral arterial stiffness and flow-mediated dilation in healthy humans. Am. J. Physiol. Regul. Integr. Comp. Physiol..

[120] Miyachi M. (2013). Effects of resistance training on arterial stiffness: A meta-analysis. Br. J. Sport. Med..

[121] Currie K.D., Dubberley J.B., McKelvie R.S., MacDonald M.J. (2013). Low-volume, high-intensity interval training in patients with CAD. Med. Sci. Sport. Exerc..

[122] Schjerve I.E., Tyldum G.A., Tjønna A.E., Stølen T., Loennechen J.P., Hansen H.E., Haram P.M., Heinrich G., Bye A., Najjar S.M. (2008). Both aerobic endurance and strength training programmes improve cardiovascular health in obese adults. Clin. Sci..

[123] Ramos J.S., Dalleck L.C., Tjonna A.E., Beetham K.S., Coombes J.S. (2015). The impact of high-intensity interval training versus moderate-intensity continuous training on vascular function: A systematic review and meta-analysis. Sport. Med..

[124] Ramírez-Vélez R., Hernández-Quiñones P.A., Tordecilla-Sanders A., Álvarez C., Ramírez-Campillo R., Izquierdo M., Correa-Bautista J.E., Garcia-Hermoso A., Garcia R.G. (2019). Effectiveness of HIIT compared to moderate continuous training in improving vascular parameters in inactive adults. Lipids Health Dis..

[125] Da Silva M.R., Waclawovsky G., Perin L., Camboim I., Eibel B., Lehnen A.M. (2020). Effects of high-intensity interval training on endothelial function, lipid profile, body composition and physical fitness in normal-weight and overweight-obese adolescents: A clinical trial. Physiol. Behav..

[126] Tabata I., Nishimura K., Kouzaki M., Hirai Y., Ogita F., Miyachi M., Yamamoto K. (1996). Effects of moderate-intensity endurance and high-intensity intermittent training on anaerobic capacity and VO_2max_. Med. Sci. Sport. Exerc..

[127] Hanssen H., Nussbaumer M., Moor C., Cordes M., Schindler C., Schmidt-Trucksass A. (2015). Acute effects of interval versus continuous endurance training on pulse wave reflection in healthy young men. Atherosclerosis.

[128] Harris R.A., Padilla J., Hanlon K.P., Rink L.D., Wallace J.P. (2008). The flow-mediated dilation response to acute exercise in overweight active and inactive men. Obesity.

[129] Johnson B.D., Padilla J., Wallace J.P. (2012). The exercise dose affects oxidative stress and brachial artery flow-mediated dilation in trained men. Eur. J. Appl. Physiol..

